# Supplementary material for: Cryopreservation of human pluripotent stem cell-derived cardiomyocytes is not detrimental to their molecular and functional properties
Source: Stem Cell Res. Author manuscript; Available in PMC 2021 Jul 23. (PMC7611364; doi:10.1016/j.scr.2019.101698)
Supplement: Supplementary data [file EMS130813-supplement-Supplementary_data.pdf]

Supplementary data to:

Cryopreservation of human pluripotent stem cell-derived cardiomyocytes is not detrimental to their molecular and functional properties

Lettine van den Brink[1], Karina O. Brandão[1], Loukia Yiangou[1], Mervyn P.H. Mol[1], Catarina Grandela[1], Christine L. Mummery[1], Arie O. Verkerk[2], Richard P. Davis[1*]

1. Department of Anatomy and Embryology, Leiden University Medical Center, 2300 RC Leiden, The Netherlands.
2. Department of Medical Biology, Amsterdam UMC, 1105 AZ Amsterdam, The Netherlands

- Corresponding author at: Leiden University Medical Center, Department of Anatomy and Embryology, Einthovenweg 20, 2300 RC Leiden, The Netherlands.

Email address: r.p.davis@lumc.nl

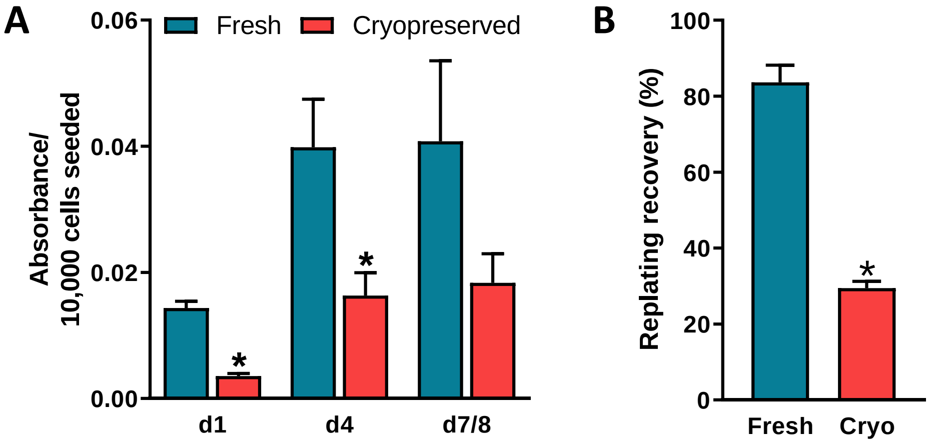

Fig. S1 Replating recovery of non-frozen and cryopreserved hiPSC-CMs derived from LUMC99 hiPSC line. A) Recovery of non-frozen and cryopreserved LUMC99 hiPSC-CMs at day 1, 4 and 7/8 post-replating (seeding density: 0.4 - 1.5 x 10^5 cells/cm^2) as determined by CCK-8 assay; n = 4-6 from 2 independent differentiations. * indicates statistical significance (day 1 $p < 0.001$, day 4 $p = 0.014$, unpaired *t*-test). **B)** Percentage of replated cells recovered 7 days after seeding (seeding density: 0.5 - 2.6 x 10^5 cells/cm^2) as determined by manual counting for non-frozen (*fresh*) and cryopreserved (*cryo*) LUMC99 hiPSC-CMs; n = 11-15 from 3 independent differentiations, respectively. * indicates statistical significance ($p<0.0001$, unpaired *t*-test).

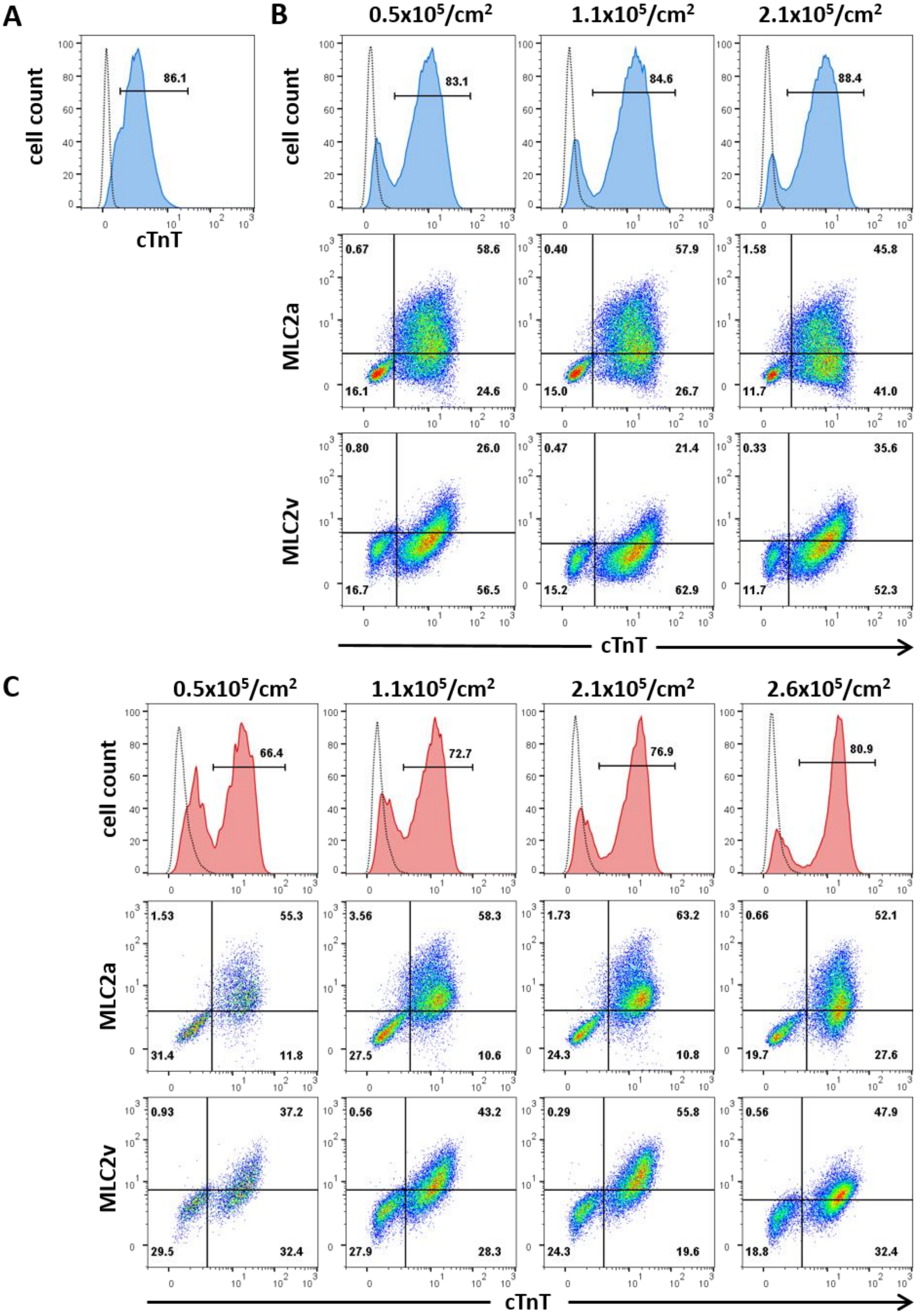

Fig. S2 Effect of replating density on cTnT, MLC2a and MLC2v expression in non-frozen and cryopreserved hiPSC-CMs derived from LUMC20. A) Histogram plot showing the percentage of hiPSC-CMs (cTnT^{+}) at d21 as determined by flow cytometry prior to replating. **B)** Replating density had little effect on the percentage of cTnT^{+} hiPSC-CMs recovered when the cardiomyocytes had not been frozen with percentages similar to those prior to replating. **C)** However for cryopreserved hiPSC-CMs, higher replating densities resulted in an increased proportion of cells expressing cTnT to similar percentages to that obtained with the freshly replated hiPSC-CMs. Although, in this representative example, a decrease in the proportion of MLC2a^{+} hiPSC-CMs was observed at the highest replating densities for both the non-frozen and cryopreserved hiPSC-CMs, in general no difference was observed in the proportion of hiPSC-CMs expressing MLC2a between low and high seeding densities (see Fig. 2G). The proportion of hiPSC-CMs expressing MLC2v did not appear to be influenced by the seeding density. Values above plots indicate the density the cells were replated. Top row depicts histogram plots of cTnT expression, while remaining rows depict bivariate density plots of MLC2a/cTnT (middle row) and MLC2v/cTnT (bottom row). Numbers inside the plots are the percentage of cells within the gated region. Dotted lines represent a control cTnT^{-} population.

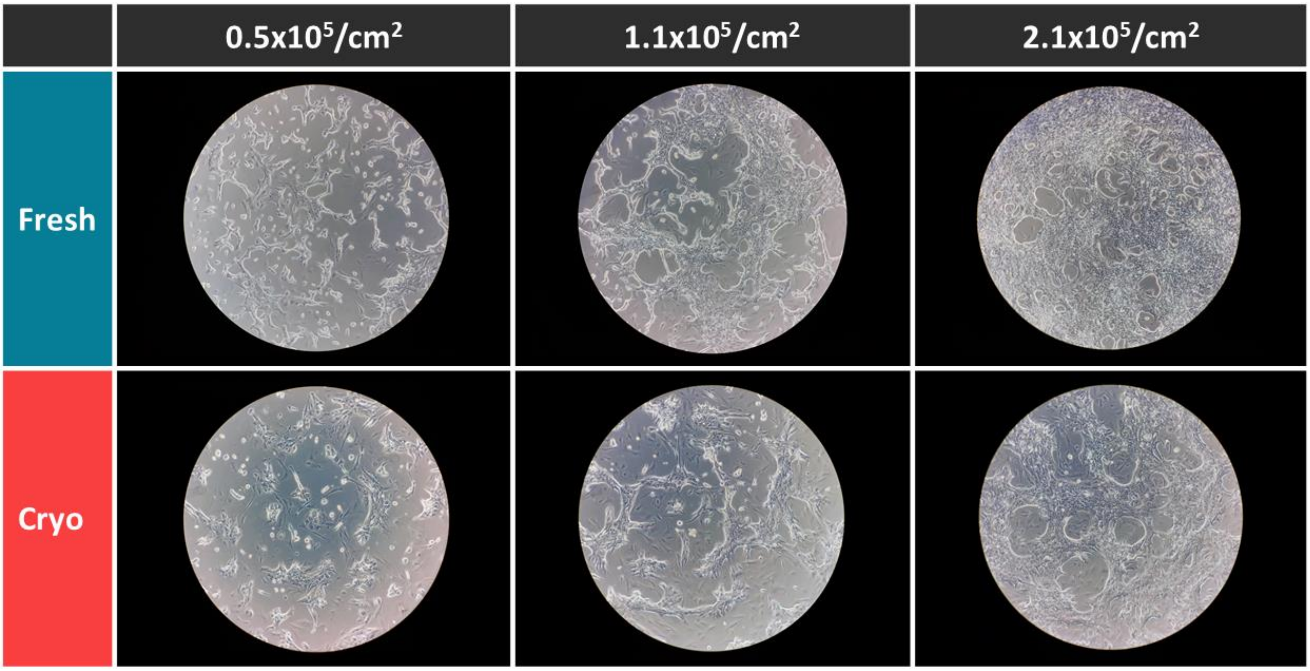

Fig. S3 Phase-contrast images of non-frozen (*fresh*) and cryopreserved (*cryo*) hiPSC-CMs derived from LUMC20 at day 7 post-replating. Values above images indicate the density the cells were replated. Original magnification, 10x.

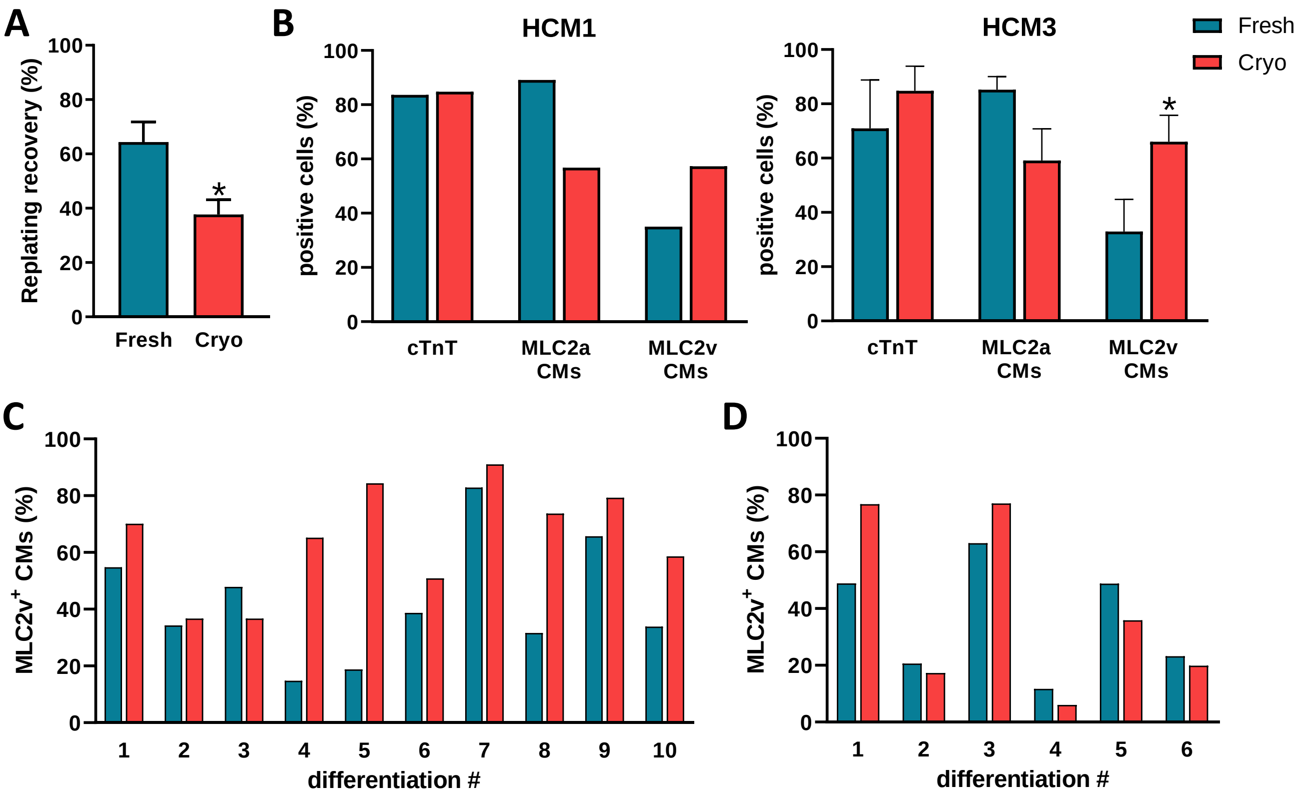

Fig. S4 Replating recovery and cardiac marker expression of non-frozen and cryopreserved hiPSC-CMs. **A)** Percentage of replated cells recovered 7 days after seeding (seeding density: 1.1 - 2.1 x 10^5 cells/cm^2) as determined by manual counting for non-frozen (*fresh*) and cryopreserved (*cryo*) HCM1 and HCM3 hiPSC-CMs; n = 5-6 from 3 independent differentiations. * indicates statistical significance (p = 0.02, unpaired *t*-test). **B)** Bar graphs showing percentage of freshly replated and cryopreserved cells from the hiPSC lines, HCM1 (*left*) and HCM3 (*right*), expressing cTnT, as well as proportion of cTnT$^+$ hiPSC-CMs expressing MLC2a or MLC2v (Seeding density: fresh, 1.1 x 10^5 cells/cm^2; cryopreserved, 2.1 x 10^5 cells/cm^2). * indicates statistical significance (p = 0.04, paired *t*-test); n = 2 independent differentiations (HCM3). **C & D)** Bar graph showing percentage of fresh and cryopreserved cTnT$^+$ hiPSC-CMs expressing MLC2v for the individual differentiations included in Figure 2H for the hiPSC lines, LUMC20 (**C**) and LUMC99 (**D**).

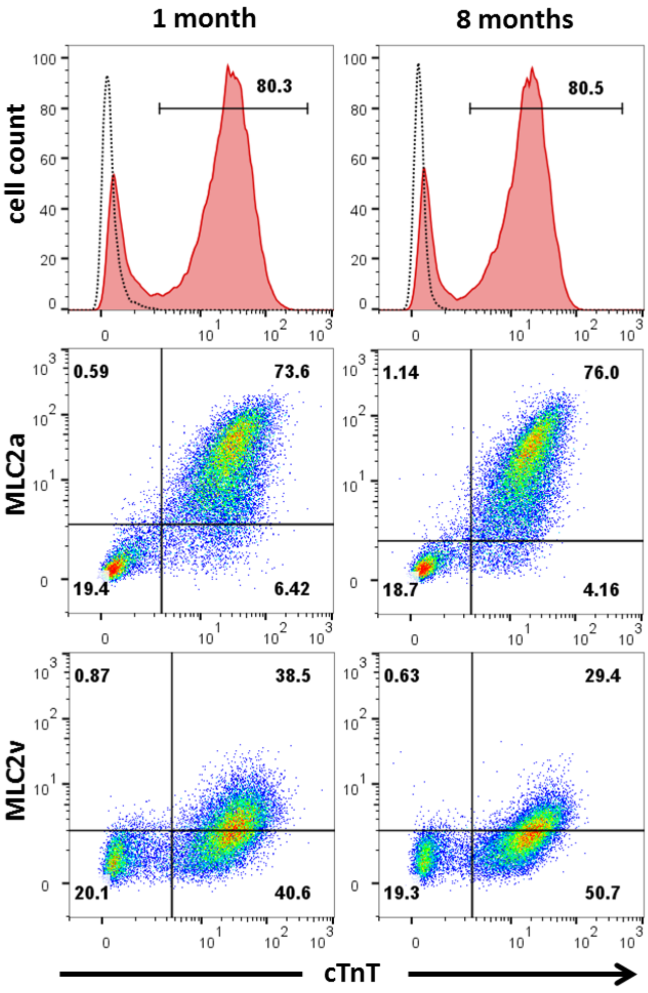

Fig. S5 No differences in cTnT, MLC2a and MLC2v expression were detected by flow cytometry analysis in LUMC20 hiPSC-CMs that had been cryopreserved for 1 month (*left column*) or for 8 months (*right column*) when replated at similar densities, 1.8 x 10^5 cells/cm^2 and 2.1 x 10^5 cells/cm^2 respectively. Top row depicts histogram plots of cTnT expression, while remaining rows depict bivariate density plots of MLC2a/cTnT (*middle row*) and MLC2v/cTnT (*bottom row*). Numbers inside the plots are the percentage of cells within the gated region. Dotted lines represent a control $cTnT^-$ population.

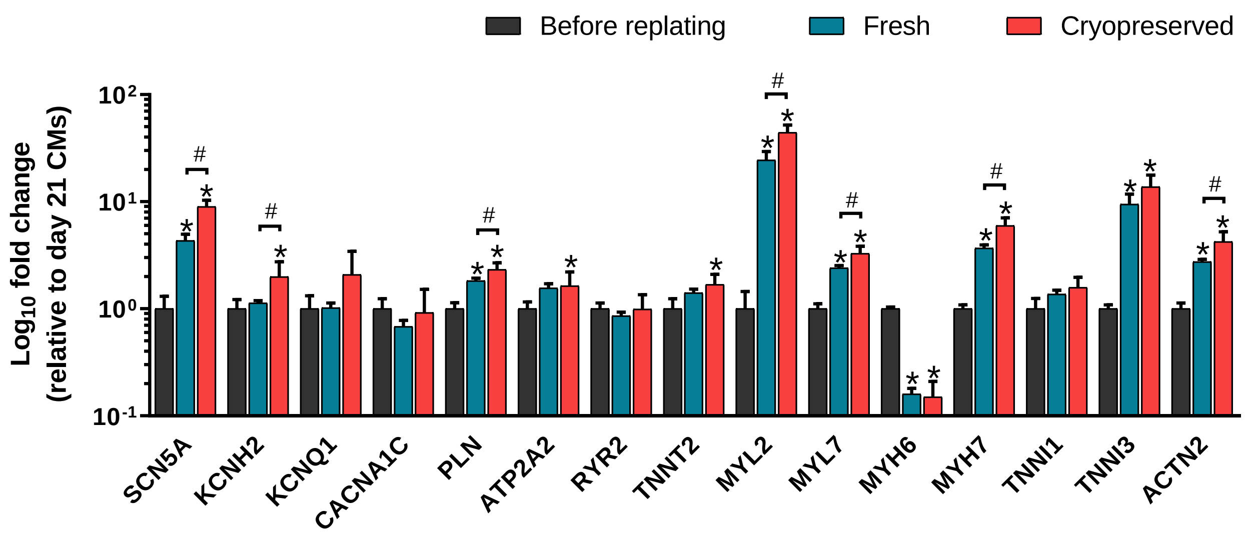

Fig. S6 qRT-PCR analysis of key genes involved in the cardiac AP, Ca^{2+} regulation and sarcomere assembly of replated non-frozen and cryopreserved LUMC20 hiPSC-CMs. Data is normalized to the housekeeping genes, *hARP* and *RLP37A*, and is relative to the expression levels in day 21 hiPSC-CMs (before replating). Data is shown as mean ± SEM and analyzed by one-way ANOVA with Tukey's post-hoc multiple comparison test. * indicates statistical significance compared to 'before replating' (fresh: *TNNI3*, p = 0.002; *PLN*, p = 0.0003; *SCN5A*, p = 0.0001; *MYL2*, *MYL7*, *MYH6*, *MYH7*, *ACTN2*, p < 0.0001; cryopreserved: *ATP2A2*, *TNNT2*, p = 0.03; *KCNH2*, p = 0.004; *SCN5A*, *PLN*, *MYL2*, *MYL7*, *MYH6*, *MYH7*, *TNNI3*, *ACTN2*, p < 0.0001); # indicates statistical significance between fresh and cryopreserved (*PLN*, p = 0.02; *KCNH2*, p = 0.01; *MYL2*, p = 0.0007; *MYL7*, p = 0.0005; *ACTN2*, p = 0.0002; *SCN5A*, *MYH7*, p < 00001); n = 3 independent differentiations.

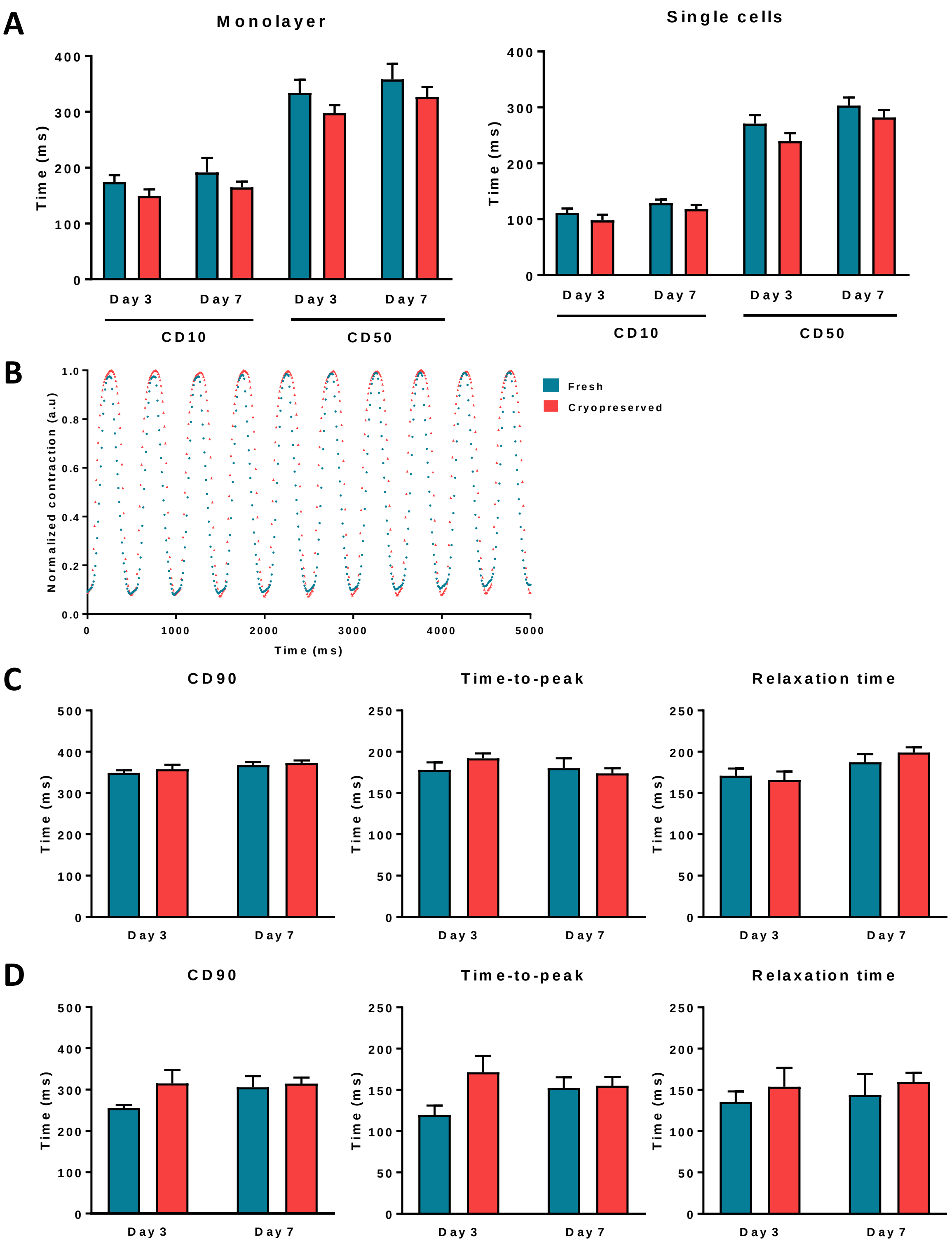

Fig. S7 A) Average data at 1 Hz for contraction duration at 10% and 50% from peak (CD10, CD50) for fresh (blue) and cryopreserved (red) LUMC20 hiPSC-CMs seeded as a monolayer (left) or as single cells (right). **B)** Representative normalized contraction traces measured at 2 Hz for fresh and cryopreserved LUMC20 hiPSC-CMs seeded as a monolayer. **C & D)** Average data at 2 Hz for CD90, time-to-peak and relaxation time for fresh and cryopreserved LUMC20 hiPSC-CMs measured in cells cultured either as monolayers (**C**) or single cells (**D**). For **C**, n = 6 and 9 (day 3); n = 7 and 9 (day 7) from 3 independent differentiations; For **D**, n = 4 and 7 (day 3); n = 5 and 10 (day 7) from 2 independent differentiations.

Supplementary Table S1. Primer sequences for qRT-PCR

Gene	Forward primer (5'-3')	Reverse primer (3'-5')
SCN5A	GAGCTCTGTCACGATTTGAGG	GAAGATGAGGCAGACGAGGA
KCNH2	CACCGCCCTGTACTTCATCT	AGGCCTTGCATACAGGTTCA
KCNQ1	TCCTGGTCTGCCTCATCTTC	AAGAACACCACCAGCACGAT
CACNA1C	CAATCTCCGAAGAGGGGTTT	TCGCTTCAGACATTCCAGGT
PLN	AGCACGTCAAAAGCTACAGAATCT	CTGATGTGGCAAGCTGCAGATC
ATP2A2	ACAATGGCGCTCTCTGTTCT	ATCCTCAGCAAGGACTGGTTT
RYR2	GCTATTCTGCACACGGTCATT	ATTTCCGTGCCACTTCCTTT
TNNT2	AGCATCTATAACTTGGAGGCAGAG	TGGAGACTTTCTGGTTATCGTTG
MYL2	TACGTTCGGGAAATGCTGAC	TTCTCCGTGGGTGATGATG
MYL7	CCGTCTTCCTCACGCTCTT	TGAACTCATCCTTGTTCACCAC
MYH6	CCAGGTCAACAAGCTTCGAG	TGTCACTCCTCATCGTGCAT
MYH7	AGTCCCAGGTCAACAAGCTG	GGGCTGAGCAGATCAAGATG
TNNI1	GTGGGTGACTGGAGGAAGAA	GTGAGCTGGGTTGGAGAAGA
TNNI3	CACCTCAAGCAGGTGAAGAAG	CAGGAAGGCTCAGCTCTCAA
ACTN2	GATGGAGCACATTCGTGTTG	TGATCCATCAGGCCATTCTT
HARP	CACCATTGAAATCCTGAGTGATGT	TGACCAGCCCAAAGGAGAAG
RPL37A	GTGGTTCCTGCATGAAGACAGTG	TTCTGATGGCGGACTTTACCG

Primers are listed as described previously (Giacomelli *et al*, 2017), except for *PLN* which was specifically designed for this study.
